# Association between illness perception and medication adherence in patients with diabetes mellitus in North Shoa, Zone: cross-sectional study

**DOI:** 10.3389/fpubh.2023.1214725

**Published:** 2023-12-19

**Authors:** Akine Eshete, Birhan Getye, Getachew Aynaddis, Bantalem Tilaye, Elda Mekonnen, Bethlehem Taye, Dereje Zeleke, Tilahun Deresse, Tewodros Kifleyohans, Yibeltal Assefa

**Affiliations:** ^1^Department of Public Health, Debre Berhan University, Debre Berhan, Ethiopia; ^2^Department of Nursing, Mizan Tepi University, Mizan Teferi, Ethiopia; ^3^School of Nursing and Midwifery, Debre Berhan University, Debre Berhan, Ethiopia; ^4^Department of Midwifery, Mizan Tepi University, Mizan Teferi, Ethiopia; ^5^School of Medicine, Debre Berhan University, Debre Berhan, Ethiopia; ^6^School of Public Health, The University of Queensland, Herston, QLD, Australia

**Keywords:** medication adherence, illness perception, patient with diabetes, perception towards medication, North Shoa Zone

## Abstract

**Background:**

Although the impact of illness perception on medication adherence is well-established, its specific influence on medication adherence in Ethiopia remains unclear. Consequently, the objective of this study was to examine the association between illness perception and medication adherence among patients with diabetes mellitus in the North Shoa Zone.

**Methods:**

An institution-based cross-sectional study was conducted from 24 May to 25 June 2022 in the North Shoa zone. The study included a random sample of 552 individuals with diabetes from four public hospitals. Data was collected and entered into Epi Data V.3.1, and analysis was performed using SPSS version 22. Descriptive statistics were used to summarize continuous variables as means with standard deviations, while categorical variables were presented as percentages. The study variables were analyzed using binary logistic regression models to assess the associations between illness perception and medication adherence. In the bivariable analysis, variables with *p*-values less than 0.20 were entered into a multivariable logistic regression model. Associations with a *p*-value ≤0.05 and an odds ratio with a 95% confidence interval were considered statistically significant.

**Results:**

The study results revealed that medication adherence was 64.4% (95% CI: 60.1, 67.9), while illness perception was 54.7% (95% CI, 41.2, 49.4). There was a significant and strong association between illness perception and medication adherence (*p* < 0.0001). In the adjusted model, the illness perception components of consequence showed a significant association with medication adherence (AOR = 3.10, 95% CI: 2.11, 4.55). Similarly, personal control (AOR = 1.77, 95% CI: 1.20, 2.61) and emotional representation of diabetes (AOR = 2.26, 95% CI: 1.54, 3.32) were also significantly associated with medication adherence in patients with diabetes.

**Conclusion:**

The findings of this study indicate a positive association between higher illness perception and increased medication adherence and practice. Therefore, when engaging in discussions about diabetic self-management, diabetes educators should employ psychoeducational approaches that take into account the illness perceptions of patients.

## Background

Diabetes is a significant global health issue, with a considerable impact on the adult population. In 2021, it was estimated that approximately 537 million adults worldwide were affected by diabetes. Projections indicate a steady rise, with an expected increase to 643 million by 2030 and 783 million by 2045. The prevalence of diabetes in Africa is also a concern, with an estimated 24 million adults affected in 2021. Alarmingly, this number is projected to rise to 55 million by 2045, highlighting the urgent need for effective prevention and management strategies in the region (1). Diabetes is increasingly becoming a significant public health challenge in Ethiopia, with a national prevalence rate of 2.8% in 2022 (2) and gradually increasing from 1% in 2000 (3) to 3.3% in 2020, according to the Report of the International Diabetes Federation (1).

The long-term social, health, and economic impacts of diabetes on individuals and countries are substantial. Consequently, treatment adherence serves as a crucial indicator of healthcare quality in the effective management of diabetes (4). Adherence to treatment refers to the voluntary cooperation of patients in following the prescribed dosage, timing, and frequency of medication as directed by healthcare professionals (5, 6).

Medication adherence plays an important role in glycemic control and prevention of complications (7) and the effectiveness of treatment also depends on adherence to recommended medication (8). Low adherence to medications in people with diabetes is significantly associated with poor glycemic control (9). Although a wide range of antidiabetic drugs is available, many patients with diabetes still fail to control blood glucose levels associated with diabetes (10). Patients’ adherence to prescribed medications is influenced by personal beliefs about chronic diseases (11). Adherence to antidiabetic medications improves with better illness perception (12). Illness perception (IP) is characterized as the patient’s implicit and common sense beliefs regarding their illness (10).

Illness perception plays a key role in the study of patients’ perceptions of their illness and significantly impacts medication adherence (13). Individuals with diabetes are more motivated to adhere to their treatment regimen when they hold the belief that their medications are effective and that their disease is well-managed (14, 15). IP plays an important role in managing adherence and is related to adherence in diabetes (16). Research findings indicate that having knowledge about one’s illness positively influences self-care behaviors, which in turn help individuals with diabetes to mitigate the consequences of the disease (14).

Hence, it is crucial to manage illness perception among individuals with diabetes as it has the potential to enhance treatment adherence. Research evidence strongly indicates a significant relationship between illness perception and treatment adherence in diabetes, resulting in improved management and control of the disease (13, 17). Patient perception is a psychological factor in diabetes that directly or indirectly influences treatment adherence, emphasizing the need for additional research to investigate the role of psychological factors in diabetes management (18). Patient perception, as a psychological aspect, should be integrated into diabetes care and effectively communicated across all levels of the healthcare system.

To the best of our knowledge, there are no studies that have investigated the relationship between illness perception and treatment adherence in Ethiopia. The hypothesis put forth is that a positive recognition of diabetes as an illness may have a favorable influence on medication adherence. Therefore, the objective of this study was to examine the association between disease perception and treatment adherence in the Northern Shoa.

## Methods

### Study design and setting

The facility-based cross-sectional study design was performed at public hospitals in the North Shoa Zone from 24 May to 25 June 2022. North Shoa is one of the thirteenth zones of the Amhara region located in northern Ethiopia. There are 24 districts, 3 municipalities, and 13 hospitals. All public hospitals have diabetes care and follow-up services.

### Subjects and sample selection

The sample size was determined using the formula for the proportion of the single population considering the proportion of medication adherence of 68% in Ethiopia (19) with a precision level of 5 and 15% for non-response and a design effect of 1.5, resulting in 552 subjects. Diabetic subjects aged ≥18 years who had been followed for more than three months were included in the study. The mean of Fasting Blood Sugar (FBS) measurements in three consecutive months was used for FBS analysis. Patients who were medically judged unable to participate in the study (e.g., acute illness, mental illness, and dementia) and patients with severe visual impairment were excluded from the study. Study participants were recruited from four hospitals, selected according to a proportional allocation among hospitals. Study participants were selected using a systematic randomization procedure. Because each patient had at least one appointment within one month, we waited up to one month for a selected study participant.

### Data measurement and tool

Medication adherence was evaluated through a set of four questions, including: Have you ever forgotten to take a prescribed medication? Do you sometimes discontinue taking your medication? Are there instances when you are not diligent about taking your medicine? And if you experience worsening symptoms after taking your medication, do you cease taking it?

The scoring method for this questionnaire focuses solely on item four. A response of “Yes” is assigned a score of 1, while a response of “No” is given a score of 0. The total score is obtained by summing the scores of all items in the questionnaire. The total score ranges from zero to four, where a score above 1 suggests low compliance, while a score of 0 indicates high compliance. The questionnaire underwent face and content validity assessment by experts, and its reliability was measured using the Cronbach’s alpha method, resulting in values ranging from 0.75 to 0.81.

The evaluation of individuals’ perception of their illness utilized the modified Illness Perception Questionnaire (IPQ-R). This questionnaire consists of seven subscales and a 38-item version (20). The instrument employed in this study consists of 38 items that assess seven domains of illness perception (20, 21). Each domain of illness perception was treated as an independent variable and examined to assess its influence on medication adherence. Participants providing their perception of illness using a 5-point Likert scale for each question within each domain. The responses were summed to calculate an average score for each domain. Furthermore, individual scores were computed for each component, and an overall score was computed to the overall level of illness perception. A higher score indicates stronger feelings associated with each aspect of illness perception (20, 22). The Cronbach’s alpha coefficients for the subscales ranged from 0.70 to 0.89.

The data collection process was carried out by four trained data collectors, all of whom held bachelor’s degrees. These collectors conducted interviews to gather the required data. Prior to data collection, both the data collectors and their supervisors underwent comprehensive training on the research objectives, questionnaire content, and the importance of maintaining confidentiality and privacy throughout the data collection process. To ensure data quality, all authors and supervisors performed daily checks to verify completeness, accuracy, and consistency of the collected data. The principal investigator closely monitored the data collection activities on a daily basis, ensuring questionnaire completeness and providing further clarification when needed. Detailed review of patients’ medical records was conducted, capturing essential information such as fasting blood sugar (FBS) levels, diabetic complications, comorbidities, and the medications being taken.

### Data management and analysis

The collected data was entered into Epi Data version 4.6 and then exported to SPSS version 25 for analysis. Descriptive analysis was utilized to present the frequency distribution of each variable in the study. Continuous variables were reported as mean ± standard deviation, while categorical variables were presented as percentages.

To examine the relationship between illness perception and medication adherence, binary logistic regression analysis was conducted. Multicollinearity was assessed using the variance inflation factor, with a threshold value of less than 10 considered acceptable. The goodness of fit of the model was evaluated using the Homer-Lemeshow test.

Variables that demonstrated a *p*-value of less than 0.20 in the bivariate analysis were included in the subsequent model analysis. The significance of the associations was determined using the *p*-value and 95% confidence interval (CI) for the odds ratios (OR).

## Results

### Socio-demographic and clinical characteristics of respondents

The study included 539 individuals diagnosed with diabetes, resulting in a high response rate of 98%. The gender distribution was nearly equal among the participants. A majority of respondents (52.1%) fell within the age range of 41 to 60 years, and a significant proportion (45.6%) were married. Moreover, a majority of the participants had received formal education.

Among all the study participants, a substantial percentage (80.1%) had fasting blood sugar (FBS) levels that were uncontrolled, surpassing 130 mg/dL. Regarding diabetic complications, 35.8% of the participants had developed chronic complications, with retinopathy (32.8%) being the most prevalent. Additionally, 145 individuals (26.9%) had comorbidities, with hypertension (51.0%) being the most frequently reported comorbidity (Table 1).

**Table 1:** Socio-demographic and clinical characteristics of respondents in North Shoa, 2022.

Variables	Category (*n* = 539)	Frequency	Percent (%)
Sex of the respondent	Male	260	48.2
	Female	279	51.8
Age (years) of the respondent	18–40	169	31.4
	41–60	246	45.6
	>60	124	23.0
Marital status of the respondent	Single	134	24.9
	Married	335	62.2
	Divorced	34	6.3
	Widowed	36	6.7
Level of education	Unable to read and write	133	24.7
	Can read and write	77	14.3
	Primary school	117	21.7
	Secondary school	105	19.5
	College and above	107	19.9
Place of residence of the respondent	Urban	222	41.2
	Rural	317	58.8
Family history of diabetes	Yes	156	28.9
	No	383	71.1
Average FBS (mg/dl)	≤130	107	19.9
	>130	432	80.1
Type of DM	Type I DM	231	42.9
	Type II DM	308	57.1
Duration of diabetes (years)	≤5	299	55.5
	>5	240	44.5
Presence of co-morbid conditions	Yes	145	26.9
	No	394	73.1
Co-morbid conditions	Hypertension	98	51.0
	Ischemic heart disease	34	17.7
	Kidney disease	14	7.3
	Dyslipidemia	37	19.3
	Other (Arthritis, HIV Thyrotoxicosis, and Asthma)	9	4.7
Presence of diabetes complications	Yes	193	35.8
	No	346	64.2
Encountered complications	Retinopathy	77	32.8
	Nephropathy	60	25.5
	Neuropathy	50	21.3
	Coronary artery disease	48	20.4

DM: diabetes mellitus; HIV: human immunodeficiency virus.

### Participant’s perception of their diabetes illness

In the study, the overall illness perception was found to be 54.7% (95%CI: 41.2–49.4%). The mean score for illness perception was 135.6 ± 8.7 SD. Among the various dimensions of illness perception, illness perception/personal control ability had the highest mean score of 20.5 ± 3.7 SD, followed by emotional illness response with a mean score of 20.8 ± 3.1 SD. On the other hand, illness identity had the lowest mean score of 8.3 ± 2.3 SD.

Table 2 presents the frequency distribution of the reported illness perceptions. The most commonly reported illness perceptions were perception of consequences (59.9%), treatment control (58.1%), and emotional expression (57.9%) (Table 2).

**Table 2:** Illness perception of respondents in North Shoa, 2022 (*n* = 539).

Variables	Category	Frequency	Percent (%)	Mean score ± SD
Identity	Low	290	53.8	8.3 ± 2.3
	High	249	46.2	
Timeline acute/chronic	Low	246	45.6	19.5 ± 2.3
	High	293	54.4	
Consequence	Low	216	40.1	19.8 ± 2.5
	High	323	59.9	
Personal control	Low	253	46.9	20.5 ± 3.7
	High	286	53.1	
Treatment control	Low	226	41.9	19.8 ± 2.0
	High	313	58.1	
Illness coherence	Low	263	48.8	14.7 ± 3.3
	High	276	51.2	
Timeline cyclical	Low	287	53.2	12.2 ± 3.8
	High	252	46.8	
Emotional representation	Low	227	42.1	20.8 ± 3.1
	High	312	57.9	
Overall illness perception	Low	244	45.3	135.6.6 ± 8.7
	High	295	54.7	

### Relationship between illness perception and medication adherence

In the study, the overall adherence to antidiabetic medication was determined to be 64.4%, with a mean of 0.67 ± 1.02 SD. Chi-square test analysis revealed a significant association between illness perception and adherence to antidiabetic medication (χ2 = 4.01; *p* < 0.05). Several domains of illness perception were found to be significantly associated with medication adherence. These domains included perception of acute/chronic timeline (*p* = 0.025), perception of diabetic consequences (*p* < 0.0001), perception of personal control (*p* = 0.001), and perception of emotional representation (*p* < 0.0001) (Table 3).

**Table 3:** Relationship of illness perception and medication adherence of diabetic patients in North Shoa 2022 (*n* = 539).

Variable	Category	Medication adherence		X2	*p*-value
		Adherent	Non-adherent		
Identity	High	166 (66.7%)	83 (33.3%)	1.057	0.304
	Low	181 (62.4%)	109 (37.6%)		
Timeline acute/chronic	High	201 (68.6%)	92 (31.4%)	4.991	0.025*
	Low	146 (59.3%)	100 (40.7%)		
Consequence	High	241 (74.6%)	82 (25.4%)	36.815	<0.0001*
	Low	106 (49.1%)	110 (50.9%)		
Personal control	High	203 (71%)	83 (29%)	11.576	0.001*
	Low	144 (56.9%)	109 (43.1%)		
Treatment control	High	201 (64.2%)	112 (35.8%)	0.008	0.927
	Low	146 (64.6%)	80 (35.4%)		
Illness coherence	High	190 (66.4%)	96 (33.6%)	1.122	0.289
	Low	157 (62.1%)	96 (37.9%)		
Timeline cyclical	High	169 (67.1%)	83 (32.9%)	1.488	0.223
	Low	178 (62%)	109 (38%)		
Emotional representation	High	226 (72.4%)	86 (27.6%)	20.973	<0.0001*
	Low	121 (53.3%)	106 (46.7%)		
Overall illness perception	High	201 (68.1%)	94 (31.9%)	4.011	0.045*
	Low	146 (59.8%)	98 (40.2%)		

### Association between only illness perception domain and medication adherence

In the adjusted model, patients who had higher perceptions of diabetic consequences were found to be three times more likely to adhere to treatment compared to other patients (adjusted odds ratio [AOR] = 3.10, 95% CI: 2.11, 4.55). Similarly, individuals with a sense of personal control over their diabetes (AOR = 1.77, 95% CI: 1.20, 2.61) and those with an emotional perception (AOR = 2.26, 95% CI: 1.54, 3.32) were more inclined to adhere to treatment (Table 4).

**Table 4:** Binary logistic regression analysis of the relationship of illness perception and medication adherence in North Shoa, 2022 (*n* = 539).

Variable	Category	Medication adherence		COR (95%CI)	AOR (95% CI)
		Adherent	Non-adherent		
Identity	High	166 (66.7%)	83 (33.3%)	1.2 (0.85, 1.72)	1.16 (0.79, 1.70)
	Low	181 (62.4%)	109 (37.6%)	1	1
Timeline acute/chronic	High	201 (68.6%)	92 (31.4%)	1.5 (1.05, 2.13)	1.38 (0.94, 2.03)
	Low	146 (59.3%)	100 (40.7%)	1	1
Consequence	High	241 (74.6%)	82 (25.4%)	3.1 (2.12, 4.40)	3.10 (2.11, 4.55)**
	Low	106 (49.1%)	110 (50.9%)	1	1
Personal control	High	203 (71%)	83 (29%)	1.9 (1.30, 2.64)	1.77 (1.20, 2.61)**
	Low	144 (56.9%)	109 (43.1%)	1	1
Treatment control	High	201 (64.2%)	112 (35.8%)	0.98 (0.69, 1.41)	0.84 (0.56, 1.25)
	Low	146 (64.6%)	80 (35.4%)	1	1
Illness coherence	High	190 (66.4%)	96 (33.6%)	1.21 (0.85, 1.72)	1.22 (0.83, 1.80)
	Low	157 (62.1%)	96 (37.9%)	1	1
Timeline cyclical	High	169 (67.1%)	83 (32.9%)	1.25 (0.87, 1.78)	1.17 (0.80, 1.72)
	Low	178 (62%)	109 (38%)	1	1
Emotional representation	High	226 (72.4%)	86 (27.6%)	2.30 (1.61, 3.30)	2.26 (1.54, 3.32)**
	Low	121 (53.3%)	106 (46.7%)	1	1

Statistically significant variables ** at *p* < 0.05 in multivariable analysis; CI: confidence interval at 95%; 1 reference variable; COR: crude odds ratio; AOR; adjusted odds ratio.

### Association of medication adherence with illness perception and background information

The final model included sociodemographic, patient-related, care-related, clinical, and medication-related variables. Variables that demonstrated a *p*-value of less than 0.20 in the bivariate analysis were considered for inclusion. After adjusting for potential confounding factors, it was observed that patients with a higher perception of diabetic consequences (AOR = 2.5, 95% CI: 1.64, 3.72), patients with good personal control over their diabetes (AOR = 1.78, 95% CI: 1.19, 2.68), and those with an emotional perception (AOR = 2.39, 95% CI: 1.59, 3.58) were more likely to adhere to their treatment (Table 5).

**Table 5:** Logistic regression analysis of medication adherence with illness perception and background information among the participants in North Shoa Zone, 2022.

Variable	Category	Medication adherence		COR (95%CI)	AOR (95% CI)
		Adherent	Non-adherent		
Family history	Yes	107 (68.6%)	49 (31.4%)	1.30 (0.88, 1.93)	1.44 (0.92, 2.27)
	No	240 (62.7%)	143 (37.3%)	1	1
Presence of co-morbid conditions	Yes	81 (55.9%)	64 (44.1%)	0.61 (0.41,0.90)	1.10 (0.60, 2.00)
	No	266 (67.5%)	128 (32.5%)	1	1
Availability	Yes	178 (73.3%)	65 (26.7%)	2.06 (1.43, 2.97)	1.99 (1.32, 3.01)**
	No	169 (57.1%)	127 (42.9%)	1	1
Counseling/education	Yes	150 (68.8%)	68 (31.2%)	1.39 (0.97, 2.00)	1.29 (0.85, 1.95)
	No	197 (61.4%)	124 (38.6%)	1	1
Age	18–40	114 (67.5%)	55 (32.5%)	2.78 (1.72, 4.49)	2.18 (1.28, 3.72)**
	41–60	180 (73.2%)	66 (26.8%)	3.65 (2.32, 5.75)	3.32 (2.00, 5.51)**
	>60	53 (42.7%)	71 (57.3%)	1	1
Duration of treatment	≤5	212 (67.1%)	104 (32.9%)	1.33 (0.93, 1.90)	0.46 (0.19, 1.13)
	>5	135 (60.5%)	88 (39.5%)	1	1
Number of drugs taken per day	One	131 (71.6%)	52 (28.4%)	2.70 (1.71, 4.28)	2.35 (1.18, 4.69)**
	Two	147 (69%)	66 (31%)	2.39 (1.54, 3.70)	2.07 (1.12, 3.80)**
	Three or more	69 (48.3%)	74 (51.7%)	1	1
Duration of diabetes	≤5	206 (68.9%)	93 (31.1%)	1.56 (1.09, 2.22)	2.26 (0.92, 5.56)
	>5	141 (58.8%)	99 (41.2%)	1	1
Timeline acute/chronic	High	201 (68.6%)	92 (31.4%)	1.50 (1.05, 2.13)	1.41 (0.94, 2.12)
	Low	146 (59.3%)	100 (40.7%)	1	1
Consequence	High	241 (74.6%)	82 (25.4%)	3.05 (2.12,4.40)	2.47 (1.64, 3.72)**
	Low	106 (49.1%)	110 (50.9%)	1	1
Personal control	High	203 (71%)	83 (29%)	1.85 (1.30, 2.64)	1.78 (1.19, 2.68)**
	Low	144 (56.9%)	109 (43.1%)	1	1
Emotional representation	High	226 (72.4%)	86 (27.6%)	2.30 (1.61, 3.30)	2.39 (1.59, 3.58)**
	Low	121 (53.3%)	106 (46.7%)	1	1

For statistically significant variables * at *p* < 0.05 in multivariable analysis. CI: confidence interval at 95%; 1 reference variable; COR: crude odds ratio; AOR; adjusted odds ratio.

## Discussion

The current study aimed to examine the relationship between illness perception and medication adherence in the North Shao Zone. The findings revealed a significant association between illness perception and adherence to antidiabetic medication (X2 = 4.01; *p* < 0.05). These results align with previous research indicating a positive relationship between illness perception and treatment adherence (13, 16, 23–26). Illness perception influences treatment adherence by affecting patients’ behavior and actions (14, 27, 28). Interpersonal problems and illness perceptions significantly influence the emotional adjustment of individuals with diabetes (29).

Although illness perception has been found to have a positive impact on treatment adherence and diabetes management, a considerable number of patients in this study (54.7%) reported suboptimal illness perception. Given the significance of illness perception, it is crucial to prioritize this aspect in educational interventions aimed at improving adherence. It is essential to integrate patient illness perception into diabetes care at all healthcare levels. Furthermore, patients with negative perceptions of their disease can benefit from practical interventions, such as psychoeducational and cognitive-behavioral approaches. By addressing illness perception comprehensively, healthcare providers can contribute to better patient outcomes in diabetes care (11).

This study aimed to examine the influence of illness perception domains on medication adherence. The results demonstrated significant associations between medication adherence and three domains of illness perception: acute/chronic timelines (*p* = 0.025), consequences (*p* < 0.0001), and emotional representation (*p* < 0.0001). The findings of this study are consistent with previous research (23, 24), reinforcing the importance of understanding illness perception in order to enhance medication adherence. The results of the study highlight the significance of gaining insights into individuals’ perceptions of their illness, as it can guide the development of strategies to improve medication adherence.

Based on the final model, medication adherence showed a positive association with illness consequences, personal control, and perception of emotional representation. Individuals with diabetes who have a comprehensive understanding of their condition are more likely to adhere to their prescribed medication. Particularly, patient-perceived illness consequences emerged as a significant factor influencing diabetes medication adherence, which is in line with previous research findings (16, 30). The influence of patient-perceived illness consequences on diabetes medication adherence can be attributed to various factors, including motivation, personal relevance, and sense of control, emotional responses, and patient-provider collaboration. Recognizing these factors can assist healthcare providers in customizing interventions and support strategies to improve medication adherence among patients with diabetes.

In this current study, patients who had a positive perception of personal diabetes control were more likely to take diabetes medication. This result is consistent with studies conducted in Saudi Arabia (16), Tripoli (24), and Iran (13). These results suggest that positive perceptions of diabetes control can promote better health outcomes. Perceived personal control is closely related to a patient’s commitment and confidence in their capacity to manage their disease and take their medications as prescribed. People are becoming more aware of their condition and health, and they feel more accountable to adhere to treatment regimens more closely. As a result, patients are confident in their ability to control their illness, which influences adherence to medications.

According to this study, a stronger perception of emotional representation was linked to a higher probability of taking diabetic medicine as prescribed. Similar results were obtained in Ghana (30), however different results were obtained in Malaysia (17). This suggests that interventions to improve emotional representation could help improve treatment adherence. Interventions could include better communication between health care providers and patients, improved patient education, and better recognition of emotional needs. In addition, we need to understand how patients perceive diabetes, because without emotional representation, treatment may not be appropriately tailored to the patient’s needs.

One of the strengths of this study is its examination of the relationship between various dimensions of illness perception and medication adherence. This highlights the need for healthcare professionals to target specific aspects of illness perception when addressing medication adherence. However, it is important to acknowledge the limitations of this study. Firstly, self-reported measures are susceptible to response bias, which may lead to an overestimation of behavioral performance. Additionally, participant recall bias regarding antidiabetic medication adherence could influence the study’s results. Moreover, the instrument used in the study should be further refined to ensure accurate and reliable data, taking into account the local context.

### The implication of the study

Research examining the association between illness perception and medication adherence in patients with Diabetes mellitus has made significant contributions to the field. By understanding how individuals perceive their illness and how it impacts their adherence to medication regimens, valuable insights have been gained for healthcare professionals, leading to the development of effective interventions. Illness perception refers to an individual’s cognitive and emotional understanding of their illness, including their beliefs, expectations, and perceived consequences. Studies have shown that patients with positive illness perceptions are more likely to adhere to their medication regimens. This includes patients who have a better understanding of the chronic nature of diabetes, believe in the effectiveness of their treatment, and feel empowered to manage their condition.

The comprehensive understanding of illness perception and its impact on patient behavior has paved the way for the development of personalized interventions that address the unique needs of each patient, ultimately improving long-term treatment outcomes. It emphasizes the importance of incorporating illness perception as a key focus in educational interventions aimed at enhancing adherence. These research findings have significant implications for clinical practice. Healthcare professionals can utilize this knowledge to tailor interventions and enhance medication adherence in patients with Diabetes mellitus. By addressing patients’ illness perceptions, correcting misconceptions through education, and fostering a sense of control and self-efficacy, healthcare providers can make strides in improving medication adherence and, subsequently, patient outcomes. Gaining insight into the influence of illness perception on patient adherence and developing appropriate strategies should be a priority for healthcare professionals.

In summary, research on the association between illness perception and medication adherence in patients with Diabetes mellitus has shed light on the psychological factors that influence adherence behaviors. This valuable knowledge has the potential to inform interventions, support patient-centered care, and ultimately enhance the management of diabetes.

## Conclusion

The current study demonstrates a positive association between higher illness perception and increased medication adherence and practice. Specifically, personal control, illness consequences, and emotional representation were identified as significant aspects of illness perception strongly linked to enhanced medication adherence.

## Data availability statement

The datasets presented in this study can be found in online repositories. The names of the repository/repositories and accession number(s) can be found in the article/supplementary material.

## Ethics statement

The studies involving humans were approved by Asrat Woldeyes Health Science Campus, Debre Berhan University, and Institutional Review Board. The studies were conducted in accordance with the local legislation and institutional requirements. The participants provided their written informed consent to participate in this study.

## Author contributions

BG and AE: conceptualization, formal analysis, project administration, and writing – original draft. AE, BG, GA, BaT, EM, BeT, DZ, TD, TK, and YA: investigation, methodology, and writing – review & editing. All authors contributed to the article and approved the submitted version.
